# Clinical Evaluation of Diode Laser as an Adjunct to Scaling and Root Planing in Patients With Chronic Periodontitis and Type II Diabetes Mellitus: A Split-Mouth Study

**DOI:** 10.7759/cureus.54178

**Published:** 2024-02-14

**Authors:** Agamjot K Lalli, Sachin Dhingra, Aeqamdeep S Lalli, Gurleen Kaur, Harinder Gupta, Sanjay Goyal

**Affiliations:** 1 Periodontology, Sri Guru Ram Das Institute of Dental Sciences & Research, Amritsar, IND; 2 Periodontology, Maulana Azad Institute of Dental Sciences, New Delhi, IND; 3 Emergency Medicine, Sub-Divisional Hospital, Batala, IND; 4 Emergency Medicine, District Hospital, Mohali, IND; 5 Periodontology, Government Dental College and Hospital, Patiala, Patiala, IND; 6 Medicine, Government Medical College, Patiala, Patiala, IND

**Keywords:** diabetes mellitus type 2, laser treatment, diode laser therapy, non-surgical periodontal therapy, chronic generalized periodontitis

## Abstract

Introduction: The primary goal of periodontal therapy is to arrest the inﬂammatory disease process which is done by non-surgical and surgical therapies in order to reduce the microorganisms. The outcome of periodontal therapy may not reveal the desired results owing to inaccessible areas for instrumentation, pathogenicity, resistance of the microorganisms, or even due to compromised host response to the treatment. Thus, adjunctive laser therapy has been proposed as a novel treatment modality in the treatment of periodontal disease.

Aim: The aim of this study was to clinically evaluate the effect of a 980 nm diode laser (DEN10B; Wuhan Gigaa Optronics Technology Co., Ltd., Wuhan, China) therapy as an adjunct to scaling and root planing in patients with chronic periodontitis and type II diabetes.

Methods: Twenty patients were divided into two groups in a split-mouth study design. Group I (Control) comprised mechanical debridement alone and Group II (test) comprised mechanical debridement followed by adjunctive laser therapy. The clinical parameters were recorded at baseline, six weeks, and three months, and the results were analyzed.

Results: There was a significant improvement in gingival and plaque index in the test group. Though there was no significant improvement in probing pocket depth and clinical attachment, the results in the test group were superior relative to the control group.

Conclusion: Non-surgical periodontal therapy with adjunctive use of diode laser is effective in the management of generalized chronic periodontitis in patients with type II diabetes which led to a significant reduction in plaque score, gingival index score, probing pocket depth, and gain in clinical attachment level.

## Introduction

Chronic periodontitis begins with microbial plaque accumulation on the tooth surface at the gingival margin thereby inducing an inﬂammatory reaction that results in the destruction of the periodontal tissues [1]. Diabetes mellitus (DM) is characterized by inherited or acquired deficiency in the production of insulin by the pancreas or by the ineffectiveness of insulin produced [2]. It is a heterogeneous group of disorders affecting the metabolism of carbohydrates, lipids, and proteins resulting in an abnormal elevation in blood glucose levels. The risk of periodontitis is increased roughly threefold in patients with DM compared with those without DM [3]. Diabetes does not result in gingivitis or periodontal defects per se, but it alters the response of the periodontal tissues to local pathogenic factors, causing a reduction in defense mechanisms and an increased susceptibility to infection [4]. A two-way relationship exists between diabetes mellitus and periodontal disease [5].

The primary goal of periodontal therapy is to arrest the inﬂammatory disease process, which is done by non-surgical and surgical therapies to reduce the microorganisms. Scaling and root planing have been shown to dramatically reduce the numbers of subgingival microorganisms thereby producing a shift in the composition of subgingival biofilm from gram-negative anaerobes to gram-positive facultative bacteria. The outcome of periodontal therapy may sometimes not reveal the desired results owing to inaccessible areas for instrumentation, pathogenicity, resistance of the microorganisms, or even due to compromised host response to the treatment. Thus, adjunctive laser therapy has been proposed as a novel treatment modality in the treatment of periodontal disease [1,6].

Thermal and photo-disruptive effects of the laser cause the complete elimination of periodontal pathogens [7]. Excellent bactericidal and detoxification effects have been achieved due to the deeper depth of penetration of lasers resulting in tissue ablation thereby making lasers one of the most promising technical modalities for nonsurgical periodontal treatment [7]. The diode laser is absorbed selectively by melanin and hemoglobin and possibly the pigments contained in pathogenic bacteria resulting in their destruction [8]. It specifically targets unhealthy tissues. When a laser tip is introduced in periodontal pockets, its ablating action removes the epithelial lining of the soft tissue walls of the pocket and inflammatory infiltrates, and low-dose radiation scatters into surrounding tissues, thus accelerating the healing process. Photobiomodulation is a photochemical reaction caused by laser in the cells, which induces tissue repair and wound healing, possibly due to increased circulation, reduced edema, and minimal pain [2].

The aim of this study was to clinically evaluate the effect of diode laser therapy as an adjunct to scaling and root planing in patients with chronic periodontitis and type II DM.

## Materials and methods

This study was conducted in the Department of Periodontology, Government Dental College & Hospital, Patiala, India. The study was approved by the Internal Review Board of Government Dental College & Hospital (IRB 4167, dated November 25, 2019) and finally approved by the Institutional Review Board of Baba Farid University of Health Sciences (approval number: BFUHS/2K19/p.TH/13229). The inclusion criteria were: age group of 30-60 years and patients with generalized chronic periodontitis and well-controlled Type II DM (based on glycated hemoglobin (HbA1c) levels according to the criterion of the American Diabetic Association), visiting the Department of Periodontology, Government Dental College & Hospital, Patiala. Patients with any other systemic conditions, immunocompromised patients, smokers, alcoholics, pregnant females, and those who had undergone an antibiotic course in the preceding six months were excluded from the study. Patients using any medicated toothpaste or antibacterial mouthwash were also excluded.

A total of 20 patients who fulfilled the above inclusion criteria were included in the study. A written informed consent was obtained from all the subjects after explaining the study procedure in detail.

Two similar teeth of posterior segments, one on either side of the arch having a pocket depth of ≤5 mm were selected. The site/surface of the selected teeth with the maximum probing pocket depth was enrolled and subjected to the following clinical parameters at baseline and at intervals of six weeks and three months after the therapy: (i) Plaque index (Quigley-Hein plaque index), (ii) Loe and Sillness Gingival index [9], (iii) Probing pocket depth (using UNC-15 Probe (Hu-Friedy Mfg. Co., LLC, Chicago, Illinois, United States) and customized acrylic stent), and (iv) Clinical attachment level (using UNC-15 Probe and customized acrylic stent).

A split-mouth, double-blind study design was used. Selected teeth/sites were randomly divided into two groups using the coin method as follows: (i) Group I (control group): mechanical debridement followed by root planing using local anesthetic spray, and (ii) Group II (test group): mechanical debridement followed by root planing using local anesthetic spray with adjunctive laser therapy.

Laser therapy

**Figure 1:**
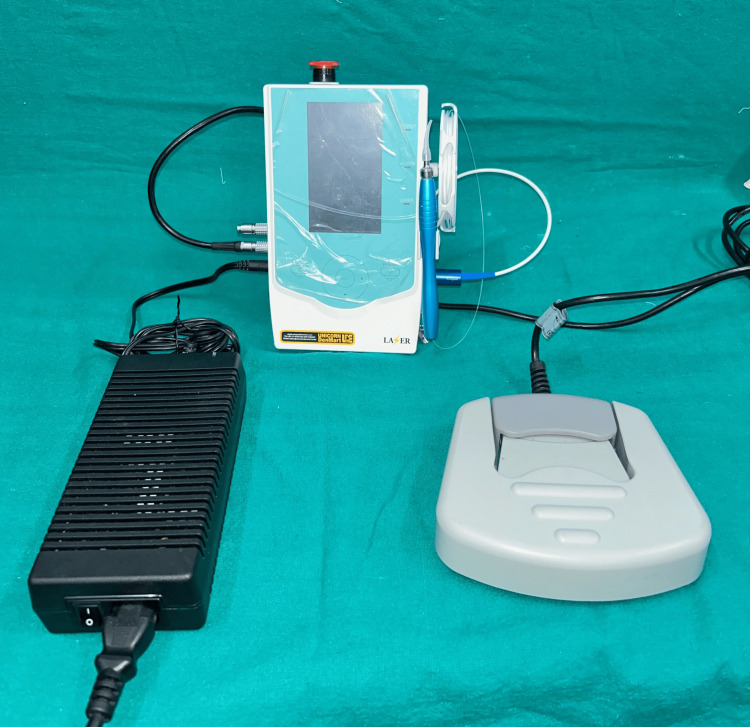
A 980 nm diode laser (Gigaa Laser DEN10B*) *Wuhan Gigaa Optronics Technology Co. Ltd., Wuhan, China

**Figure 2:**
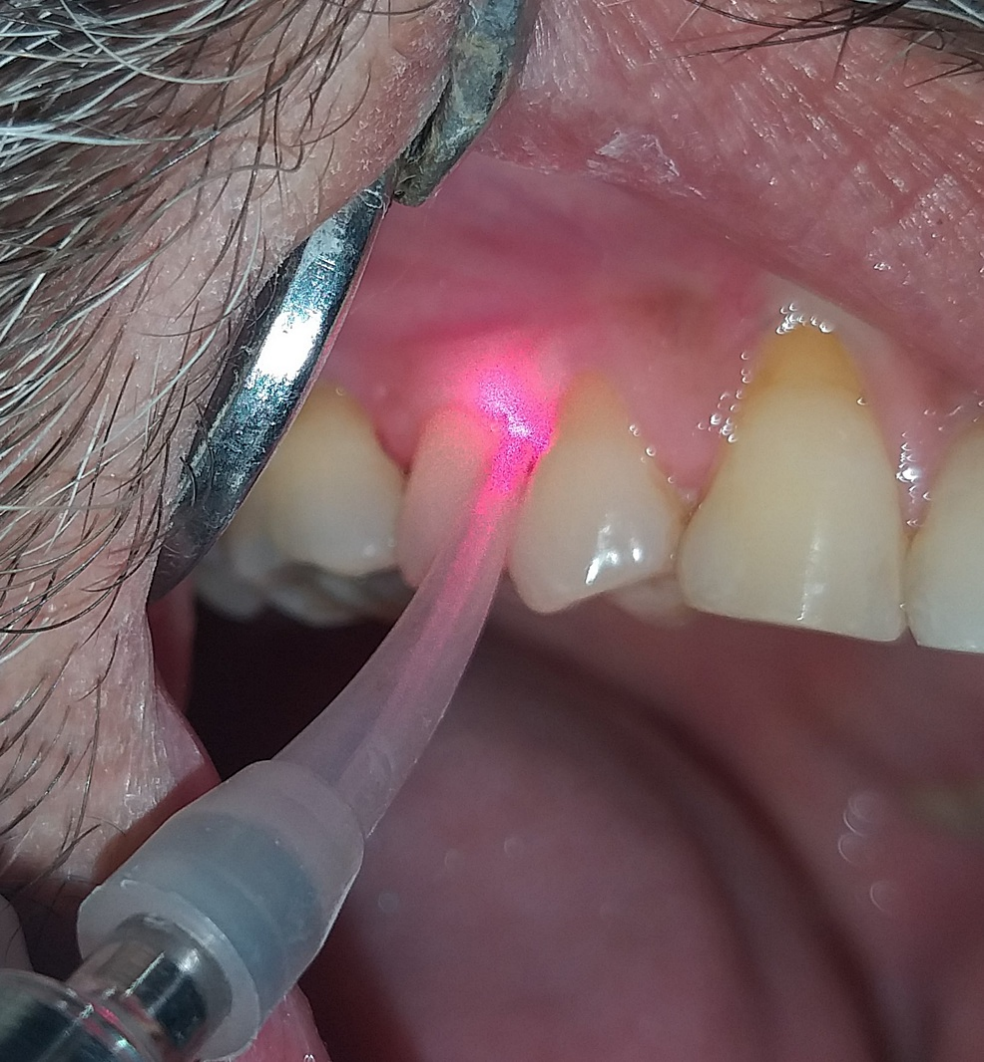
The tip of the laser or optical fiber is inserted into the periodontal pocket base in parallel alignment with the root surface.

A 980 nm diode laser (DEN10B; Wuhaan Gigaa Optronics Technology Co. Ltd., Wuhan, China) at a power of 1.5 W in pulse mode was used by the principal investigator (Figure 1). The tip of the laser or optical fiber was inserted into the periodontal pocket base in parallel alignment with the root surface (Figure 2). The laser was then activated and the fiber was moved slowly from apical to coronal in a sweeping motion during the laser light irradiation. This was done from mesial to distal at buccal aspect for 10 seconds and distal to mesial at lingual aspect for 10 seconds; reaching a total of 20 seconds for each tooth. The site was then irrigated with normal saline.

Statistical analysis

Statistical analyses were performed using IBM SPSS Statistics for Windows, Version 22.0 (Released 2013; IBM Corp., Armonk, New York, United States). The Mann-Whitney U test and Wilcoxon signed-rank test were used for comparing parameters between the test and control groups. A p-value <0.05 was regarded as statistically significant.

## Results

As shown in Table 1, the mean site-specific plaque index score in Group I was 2.43 ± 0.52 at baseline, 1.95 ± 0.51 at six weeks, and 1.80 ± 0.52 at three months postoperatively, and in Group II, it was 2.50 ± 0.56 at baseline, 1.90 ± 0.53 at six weeks, and 1.70 ± 0.47 at three months postoperatively. There was a greater reduction in the mean site-specific plaque index score in Group II as compared to Group I. As shown in Table 2, the difference in the mean reduction in plaque index score in Group I and Group II between baseline and 6 weeks was -0.13±0.69 (non-significant; p=0.181), between baseline and three months was -0.18±0.77 (non-significant; p=0.189), and between six weeks and three months was -0.05±0.49 (non-significant; p=0.513).

**Table 1:** The mean plaque index score in Group I and Group II at different intervals

Plaque Index	Group I	Group II
Baseline	2.43±0.52	2.50±0.56
6 weeks	1.95±0.51	1.90±0.53
3 months	1.80±0.52	1.70±0.47

**Table 2:** The mean change in plaque index score in Group I and Group II between different intervals

Time	Group	N	Mean change	Mean Difference	Z-value	p-value	Significance
Baseline to 6 weeks	I	20	0.48±0.38	-0.13±0.69	-1.338	0.181	Non-significant
	II	20	0.60±0.31				
Baseline to 3 months	I	20	0.63±0.36	-0.18±0.77	-1.314	0.189	Non-significant
	II	20	0.80±0.41				
6 weeks to 3 months	I	20	0.15±0.26	-0.05±0.49	-0.655	0.513	Non-significant
	II	20	0.20±0.25				

As shown in Table 3, the mean gingival index score of Group I was 2.53±0.35 at baseline, 1.86±0.41 at six weeks, and 1.51±0.52 at three months postoperatively, and in Group II, it was 2.51±0.33 at baseline, 1.66±0.41 at six weeks, and 1.10±0.42, and three months postoperatively. There is a greater reduction in the mean gingival index score in Group II as compared to Group I. As shown in Table 4, the difference of mean reduction in the gingival index score of Group I and Group II between baseline and six weeks was -0.19±0.65 (non-significant; p=0.119), between baseline and three months was -0.40±0.76 (significant; p=0.002), and between six weeks and three months was -0.21±0.75 (non-significant; p=0.155).

**Table 3:** The mean gingival index score in Group I and Group II at different intervals

Gingival Index	Group I	Group II
Baseline	2.53±0.35	2.51±0.33
6 weeks	1.86±0.41	1.66±0.41
3 months	1.51±0.52	1.10±0.42

**Table 4:** The mean change in gingival index score in Group I and Group II between different intervals

Time	Group	N	Mean change	Mean Difference	Z-value	P-value	Significance
Baseline to 6 weeks	I	20	0.66±0.37	-0.19±0.65	-1.559	0.119	Non-significant
	II	20	0.85±0.27				
Baseline to 3 months	I	20	1.01±0.33	-0.40±0.76	-3.047	0.002	Significant
	II	20	1.41±0.42				
6 weeks to 3 months	I	20	0.35±0.34	-0.21±0.75	-1.423	0.155	Non-significant
	II	20	0.56±0.40				

As shown in Table 5, the mean probing pocket depth of Group I was 4.15±0.59 at baseline, 2.55±0.89 at six weeks, and 2.30±0.73 at three months postoperatively, and in Group II, it was 4.20±0.62 at baseline, 2.35±0.99 at six weeks, and 2.00±0.92 at three months postoperatively. As shown in Table 6, the difference in mean reduction in probing pocket depth of Group I and Group II between baseline and six weeks was -0.25±0.04, between baseline and three months was -0.35±0.03, and between six weeks and three months was -0.10±0.02 and was found to be non-significant at all time intervals (p>0.05).

**Table 5:** The mean probing pocket depth in Group I and Group II at different intervals.

Probing Pocket Depth	Group I	Group II
Baseline	4.15±0.59	4.20±0.62
6 weeks	2.55±0.89	2.35±0.99
3 months	2.30±0.73	2.00±0.92

**Table 6:** The mean difference in probing pocket depth in Group I and Group II between different intervals

Time	Group	N	Mean change	Mean Difference	Z-value	P-value	Significance
Baseline to 6 weeks	I	20	1.60±0.82	-0.25±0.04	-0.817	0.478	Non-significant
	II	20	1.85±0.81				
Baseline to 3 months	I	20	1.85±0.67	-0.35±0.03	-1.519	0.165	Non-significant
	II	20	2.20±0.77				
6 weeks to 3 months	I	20	0.25±0.44	-0.10±0.02	-0.681	0.602	Non-significant
	II	20	0.35±0.49				

 As shown in Table 7, the mean clinical attachment level in Group I was 2.95±0.76 at baseline, 1.90±0.79 at six weeks, and 1.75±0.79 at three months postoperatively, and in Group II, it was 2.95±0.69 at baseline, 1.75±0.79 at six weeks, and 1.45±0.99 at three months postoperatively. As shown in Table 8, the difference in the mean reduction in clinical attachment level of Group I and Group II between baseline and six weeks was -0.15±0.02, between baseline and three months was -0.30±0.03, and between six weeks and three months was -0.15±0.02 and was found to be non-significant (p>0.05) at all time intervals.

**Table 7:** The mean clinical attachment level score in Group I and Group II at different intervals

Clinical Attachment Level	Group I	Group II
Baseline	2.95±0.76	2.95±0.69
6 weeks	1.90±0.79	1.75±0.79
3 months	1.75±0.79	1.45±0.99

**Table 8:** The mean difference in clinical attachment level score in Group I and Group II between different intervals

Time	Group	N	Mean change	Mean Difference	Z-value	P-value	Significance
Baseline to 6 weeks	I	20	1.05±0.39	-0.15±0.02	-1.060	0.289	Non-significant
	II	20	1.20±0.52				
Baseline to 3 months	I	20	1.20±0.52	-0.30±0.03	-1.544	0.123	Non-significant
	II	20	1.50±0.69				
6 weeks to 3 months	I	20	0.15±0.37	-0.15±0.02	-1.122	0.262	Non-significant
	II	20	0.30±0.47				

## Discussion

Periodontitis is characterized by the destruction of the periodontal tissues resulting in loss of connective tissue attachment, loss of alveolar bone, and the formation of pathological pockets around the diseased teeth. DM is characterized by altered glucose tolerance or impaired lipid and carbohydrate metabolism [10]. The association between periodontal disease and DM has been explored in several studies over the years and it has been observed that periodontal disease is more prevalent and more severe in individuals with DM as compared to those without [3,11,12]. Indeed, periodontitis is recognized as the "sixth complication" of diabetes [12]. The use of laser as an adjunct to periodontal therapy in individuals who have systemic diseases, such as DM, and are more susceptible to progressive periodontitis, has not been addressed much in clinical investigations. Therefore, the aim of present study was to clinically evaluate the effect of diode laser therapy as an adjunct to scaling and root planing in patients with chronic periodontitis and type II DM.

The aim of periodontal treatment is to restore the biological compatibility of periodontally diseased root surfaces for subsequent attachment of periodontal tissues to the treated root surface which is done by debridement of the diseased root surface, usually performed by mechanical scaling and root planing using manual or power-driven instruments. However, complete removal of bacterial deposits and their toxins from the root surface and within the periodontal pockets is not necessarily achieved with conventional therapy. The areas such as furcations, concavities, grooves, and distal sites of molars are inaccessible to instrumentation. Although systemic and local antibiotics are occasionally administered into periodontal pockets for the purpose of disinfection, the frequent use of antibiotics poses a potential risk of producing resistant microorganisms. Therefore, the development of novel systems as well as further improvement of currently used mechanical instruments for scaling and root planing is required.

As lasers can achieve excellent tissue ablation with strong bactericidal and detoxiﬁcation effects, and also reach sites that cannot be accessed with the help of conventional mechanical instrumentation, they are one of the most promising modalities for nonsurgical periodontal treatment. The adjunctive or alternative use of lasers along with conventional tools may facilitate treatment and has the potential to improve tissue healing [13]. It also helps reduce the inflammation in periodontal pockets when used along with scaling [14]. DM is also associated with impaired wound healing. Pro-inflammatory cytokines like interleukin (IL)-1β, tumor necrosis factor-alpha (TNF-α), and IL-6 are elevated in DM resulting in a prolonged inflammatory phase. Laser therapy has been reported to accelerate wound healing and decrease these inflammatory cytokines [15]. Diode laser promotes angiogenesis, augments growth factor release, and prevents root surface ablation [16].

The mean reduction in site-specific plaque index score between baseline and at intervals of six weeks and three months was significant in control and test groups, similar to the studies conducted by Kreisler et al. [17], Badeia et al. [18], and Berakdar et al. [19]. This can be attributed to the removal of local factors and further patient compliance in maintaining proper oral hygiene. Similar observations have been reported by Elevarasu et al. [2] and Koçak et al. [20] with the adjunctive use of laser therapy in diabetes patients. On intergroup comparison, the difference in the mean reduction in plaque index score was found to be statistically non-significant at all time intervals (p>0.05), similar to the studies conducted by Kreisler et al. [17], Dukić et al. [21], and Chondros et al. [22].

The mean reduction in gingival index score between baseline and at intervals of six weeks and three months was significant in both groups, similar to the studies of Zare et al. [16] and Badeia et al. [18], who suggested that this could be attributed to the removal of local factors and reduction in the severity of inflammation. On intergroup comparison, the mean reduction in gingival index score after three months was significant (p=0.002), as was seen in the study conducted by Caruso et al., who suggested that the reduction in periodontal inflammation may be related to the reduction of prostaglandin E2 (PGE2) levels due to the effects of laser [23].

Diode laser therapy benefits the periodontium directly by eliminating pathogens and disinfecting the pockets and indirectly by reducing the hyper-inflammatory status of diabetes patients [2]. In a study conducted by Kocak et al., scaling and root planing with and without laser treatment significantly reduced GCF levels of IL-6 in type 2 DM patients, thereby reflecting a great transient reduction in inflammatory molecules after treatment [20]. Laser therapy has been observed to produce anti-inﬂammatory, biostimulatory, and analgesic effects. The anti-inﬂammatory effect and edema reduction can partially be explained by the normalized homeostasis in tissue metabolism and inhibition of mast cell degranulation [24].

The mean reduction in probing pocket depth between baseline and at intervals of six weeks and three months was significant in both groups in the current study. This can be attributed to the fact that the attachment between the tooth and the gingival tissues after periodic root planing, soft tissue curettage is primarily accomplished by the formation of a long junctional epithelium that results in increased resistance of tissues against the penetration of periodontal probes [25]. The reduction in probing depth in the test group can be attributed to the disinfecting thermal effect of laser on bacteria that is basically limited to the root surface. The laser light not only eliminates bacteria but also inactivates bacterial toxins diffused within the root cementum based on the absorption of radiation by tissue (thermal effect)[2,6,18,20]. On comparison between the control and test groups, the difference in a mean reduction in probing pocket depth was found to be non-significant at all intervals (p>0.05), similar to the studies conducted by Ribeiro et al. [26], Miteva et al. [27], and Koçak et al. [20].

Laser induces local vasodilation, improving the circulation of fluids, and providing an anti-inflammatory effect. Moreover, the biostimulation of tissues leads to the acceleration of the healing process due to cell proliferation and induces changes in the physiological activity of cells [26]. The reduction of periodontal pockets can be attributed to the retraction of periodontal tissues [21]. Increased pocket depth in periodontitis patients is because the tip of the probe passes through the inflamed tissues to stop at the most coronal intact fibers. Following periodontal therapy pocket depth is decreased because of a reduction in inflammation, which results in decreased penetrability of the gingival tissues by the probe [2].

The mean reduction in clinical attachment level in the control group was significant between baseline and after six weeks. During the healing phase after scaling and root planning, it was observed that there was a gradual replacement of the inflammatory infiltrate and an increased number of capillaries present in the gingival connective tissue with that of more collagen-rich tissues [28]. Moreover, it has been observed that if oral hygiene is performed properly, marginal gingival fibrosis may form, thus adding to increased resistance of tissues against probe penetration. Similar results were reported in the studies conducted by da Cruz et al. [29], Christgau et al. [30], and Auyeung et al. [31] to evaluate the effectiveness of non-surgical periodontal therapy in diabetes patients. The mean reduction in clinical attachment level in the test group of the current study was significant at all time intervals. This is because laser application facilitates the removal of epithelium from the periodontal pockets enabling true periodontal regeneration. This is in accordance with the studies done by Elevarasu et al. [2], Badeia et al. [18], and Koçak et al. [20].

On intergroup comparison in the current study, the difference in the mean reduction in clinical attachment level was found to be non-significant (p>0.05) at all intervals, similar to studies conducted by Miteva et al. [27] and Kocak et al. [20]. An in vitro study conducted by Soares et al. concluded that laser therapy induces acceleration of mitotic processes within the irradiated tissues, as reported by the response of human periodontal ligament stem cell (hPDLSC) to laser therapy, with the observation of an ascending growth curve at the doses tested and a cumulative effect over time that may lead to gain in clinical attachment level [32].

Though there were no significant results between both groups in terms of probing pocket depth and clinical attachment level, the results were relatively superior in the test group as compared to the control group. In contrast, studies conducted by Kreisler et al. [17] and Elevarasu et al. [2] have shown significant improvement in probing pocket depth and clinical attachment level between the test and control groups.

It was observed that the diode laser was well tolerated by patients with no unusual finding with regard to postoperative healing and no adverse effects were elicited in any of the cases.

Limitations and recommendations

There were limitations to this study. The study was single-centric with a small sample size. The follow-up period was short. No microbiological evaluation was done to affirm the antibacterial action of diode laser therapy. A long-term multicentric randomized, controlled clinical trial with a larger sample size should be carried out to affirm the observations of this study.

## Conclusions

Within the limits of the study, it may be concluded that the non-surgical periodontal therapy is effective in the management of generalized chronic periodontitis in patients with type II DM, and can lead to a significant reduction in plaque score, gingival index score, probing pocket depth, and gain in clinical attachment level. The adjunctive use of diode laser demonstrated significantly better results as compared to scaling and root planing alone.

Therefore, diode lasers may be safely used as an adjunct to scaling and root planing in the management of chronic periodontitis. However, long-term, multicenter, randomized, controlled clinical trials should be carried out to affirm the observations of this study.
